# Practice and perceptions of postpartum care in Brunei Darussalam indigenous communities: a cross-sectional study

**DOI:** 10.1537/ase.250825

**Published:** 2025-11-14

**Authors:** Aziah Muhamad, May P.Y. Goh, Rahayu S. Sukri, Mohamed A. Majid, Norhayati Ahmad

**Affiliations:** 1 Environmental and Life Sciences Program, Faculty of Science, Universiti Brunei Darussalam, Brunei Darussalam, Jalan Tungku Link BE1410, Brunei Darussalam; 2 Herbal Research Group, Universiti Brunei Darussalam, Brunei Darussalam, Jalan Tungku Link BE1410, Brunei Darussalam; 3 Institute for Biodiversity and Environmental Research, Universiti Brunei Darussalam, Brunei Darussalam, Jalan Tungku Link BE1410, Brunei Darussalam

**Keywords:** postpartum, indigenous, traditional, Brunei, herbal

## Abstract

Southeast Asia has a rich heritage of postpartum customs, many of which are gradually being forgotten and replaced by modern allopathic practices. This is particularly true in Brunei Darussalam where documentation of postpartum customs is lacking. This study explores the postpartum traditions and practices of three indigenous groups in Brunei—Kedayans, Brunei Malays, and Lun Bawangs. A qualitative study was conducted in the form of interviews at market areas and homes of the respondents, primarily targeting the Kedayan, Brunei Malay and Lun Bawang indigenous groups. Data were collected from 57 respondents, including traditional medical practitioners, elderly villagers knowledgeable in traditional medicine, postnatal caregivers, and parents of young children. The respondents were interviewed with predesigned questions about their knowledge of, and experience with, traditional postpartum practices and herbal plant preparations. Our study revealed similarities between the plants and practices adopted by the indigenous groups, especially between the Malays and Kedayans. The postpartum practices of the three groups can be generally categorized into the following: confinement period, ‘mother roasting,’ baths, massages, stomach binding (*berbarut*), topical applications, dietary prescriptions and restrictions (*pantang*), herbal drinks, breast care, healing of the delivery wounds, and removal of stitches. Behavioural taboos during the postnatal period and trends among younger generations were also discussed. The traditional postpartum practices of the Bruneian indigenous groups are unique and represent a significant part of Bruneian custom and culture. It is imperative to document and promote beneficial customary postpartum practices to preserve the indigenous traditions of Brunei.

## 1. Introduction

The physical, physiological, and psychological changes women experience during the postpartum period can be drastic and overbearing and thus extensive care is crucial for restoring maternal health (Power et al., 2022). In many cultures, the postpartum period is considered perilous due to the increased susceptibility of new mothers to illnesses (Bazzano et al., 2020). Appropriate postpartum care is believed to heal the body of a mother as she nourishes her infant. Moreover, various cultures claim that lack of postpartum care results in detrimental health problems in later years (Power et al., 2022). Western (allopathic) or ‘modern’ postpartum care differs distinctly from traditional postpartum treatments whereby technological interventions are commonly incorporated in the former to meet the immediate physical health and needs of mothers, whereas the latter adopts a more holistic approach that is typically practised over an extended period of time following childbirth to heal and restore the physiological balance in the mother’s body (Yeh et al., 2014; Ab Ghaffar et al., 2021).

From a biocultural perspective (Wiley and Allen, 2020), postpartum traditions—such as confinement periods, dietary restrictions, and herbal treatments—can be understood as adaptive responses that address both the physiological needs of postpartum mothers and the sociocultural expectations placed upon them. These practices reflect local strategies to support maternal recovery and prevent illness during a vulnerable period, aligning with Wiley and Allen’s broader discussion of how health and healing are shaped by the interplay between biological imperatives and cultural logics across human societies.

Drawing from the ethnobiological theme identified by Ellen (2006), which highlighted how indigenous knowledge systems are rooted in ethnobotanical classifications and symbolic meanings passed down across generations, specific traditional postpartum practices such as the use of medicinal plants, herbal baths, or dietary restrictions are not arbitrary but embedded in a culturally coherent understanding of bodily balance and recovery. These practices represent not merely tradition but an ethnoscientific system grounded in generations of observation, experimentation, and adaptation, which results in a more holistic approach to postpartum care (Ellen, 2006).

Notably, traditional postpartum methods involve the extensive use of medicinal plants in the preparation of herbal baths, postpartum meals, and medicines (Boer and Lamxay, 2009). Zingiberaceae, Lauraceae, Umbelliferae, Piperaceae, and Leguminosae are families of some common plants used in postpartum care across numerous Asian countries, including but not limited to China, Korea, Pakistan, Nepal, Thailand, Vietnam, Lao, Cambodia, Malaysia, and Indonesia (Jordaan, 1985; Anderson, 1993; Muhamad and Mustafa, 1994; Handayani et al., 2001; Thi 2004; Dennis et al., 2007; Poulsen, 2007; Boer and Lamxay, 2009; Jamal et al., 2011; Lamxay et al., 2011).

For instance, decoction of plants such as *Blumea balsamifera* (Ngai camphor or *sambong*), *Cymbopogon citratus*, and *Alpinia galangal* (galangal or *lengkuas*) are used in herbal baths by the people of Lao and northeast Thailand (Poulsen, 2007; Boer et al., 2011). Other hill tribes from northern Thailand, such as the Akha people, reportedly consume an infusion of *Argyreia wallichii*, *Codonopsis javanica*, *Elephantopus scaber*, or *Zygostelma benthamii* to stimulate lactation, whereas other ethnic groups from the same region, including the Hmong, Lahu, and Karens tribes, take herbal chicken soups boiled with root shavings of *Drega volubilis* or *Zingiber* spp. (Anderson, 1993). Interestingly, the people from northern Vietnam (Thi, 2004) and Indonesia (Handayani et al., 2001) share the same practice of consuming *Sauropus androgynous* for the purpose of lactation. Apart from improving lactation, these herbal tonics also aid lochia removal and uterus contraction, and are especially recommended for young mothers to promote rejuvenation and ‘warm up’ their postpartum bodies, which are perceived to be in a ‘cold’ state following the loss of energy during childbirth (Thi, 2004; Dennis et al., 2007; Raven et al., 2007; Boer and Lamxay, 2009).

In Madura, a unique herbal ointment called *parem* prepared from a mixture of *Cananga odorata*, *Alpinia galanga*, and *Hibisicus abelmoschus*, which is rubbed and secured onto the mother’s abdomen with a broad waistband, is believed to help restore their pre-pregnancy figure (Jordaan, 1985). A similar practice is also observed in Malaysia, with the use of *kapur sirih* (calcium carbonate) dissolved in *Citrus aurantifolia*, or a mixture of *Cuminum cyminum*, *Languas galanga*, *Alpinia conchigera*, *Nigella sativa*, and *Zingiber officinale* (ginger) (Muhamad and Mustafa, 1994; Dennis et al., 2007). Such techniques are also reported for the postpartum rituals of other countries such as Thailand, Vietnam, Cambodia, Mexico, and Guatemala (Dennis et al., 2007); however, the plants used in these countries have not been described. According to Malay culture, a coconut oil concoction of spices and roots massaged onto the mother’s stomach, followed by compression of a hot oval granite stone wrapped in the leaves of *Cassia* sp., *Morinda* sp., and *Melastoma malabathricum* at the lower abdomen and around the waist is also common postpartum practice to ameliorate blood circulation and reposition the uterus.

The rich tradition and customs of Malay society are evident through anthropological studies whereby the use of plants was found to be intimately intertwined with their lives and customary rituals (Nasution et al., 2020; Susandarini et al., 2021; Mohamad et al., 2022). This applies to the Malay communities of Brunei Darussalam (Department of Agriculture, 2000; Alli, 2005; Abdul-Mumin, 2016; Kamsani et al., 2020; Mohamad et al., 2022). Although some studies have documented the use of herbal plants in the form of ethnomedicinal treatments in Brunei Darussalam, little research has been conducted on the postpartum practices of Brunei Malays (Abdul-Mumin, 2016; Kamsani et al., 2020; Mohamad et al., 2022). Therefore, this study focuses on compiling the customary postpartum practices and medicinal plants used among three indigenous groups in Brunei Darussalam; Kedayans, Brunei Malays, and Lun Bawangs. This documentation is intended to serve as a means to preserve and disseminate the traditional knowledge of these three indigenous groups in Brunei.

## 2. Subjects and Methods

### 2.1 Study design

This cross-sectional study was conducted between January 2013 and mid-July 2013, in the homes of the respondents and also in marketplaces. The study was conducted in the form of interviews that were targeted at focus groups from the Kedayan, Brunei Malay, and Lun Bawang indigenous groups.

Because the present study involved cultural knowledge and practices of three indigenous groups in Brunei, ethical clearance was obtained prior to conducting the study (see Ethics approval statement). The study was carried out in compliance with the ethical guidelines established by the University Research Ethics Committee.

#### 2.1.1 Inclusion criteria

**Figure 1.**
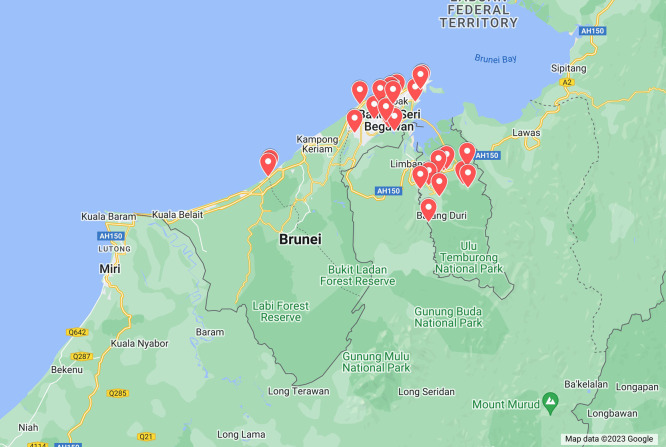
Map location of the 25 villages covered across three of the districts of Brunei Darussalam.

This study covered a total of 25 villages, distributed across three districts in Brunei Darussalam (Figure 1). The face-to-face interviews were conducted with (1) traditional medical practitioners, (2) village elders recognized for their knowledge of traditional medicine (above 50 years of age), (3) postnatal caregivers, and (4) parents with children of about four years old or younger.

The traditional villages were carefully selected to minimize potential dilution of cultural beliefs in the targeted respondents—traditional villages are rapidly disappearing and being replaced by new resettlement areas with more diverse ethnic populations. The villages inhabited by the three indigenous groups of interest were identified based on the following criteria:

1. Inhabited by the indigenous group over a reasonable time period.

2. Inhabited by a sizable concentration of the indigenous groups.

3. All respondents consented to participate in the study.

Information on the first two criteria was primarily derived from anecdotal accounts and general local knowledge, including input from village heads and community members familiar with the demographic and historical context of the area.

#### 2.1.2 Interview structure

The interviews were conducted in Brunei Malay language for ease of communication as it is the most common language locally spoken. The interviews were initiated with an introduction of the research study and aims. The participants were then shown an information sheet regarding the study in both English and Malay, and formal consent was obtained in print before the interviews proceeded. During the interview, the respondents were asked a set of predesigned questions concerning the practices and plants utilized in the traditional postpartum treatment rituals and their responses were recorded accordingly. Follow-up questions elicited further information such as the plant parts used, method of use, and dosage information.

### 2.2 Plant collection and identification

The vernacular names of the plants, along with their botanical features and place of collection, were recorded. Samples were collected from the respondents’ residential compound or purchased from the Kianggeh or Temburong market. Spices mentioned by the respondents were acquired from spice shops, or locally known as *kedai rempah*, and were identified according to literature. Identification and usages of the collected and procured plants were confirmed with the assistance of knowledgeable respondents.

Voucher specimens of the collected plant samples were prepared and submitted to the Brunei National Herbarium (BRUN) with the assistance of an experienced botanist.

### 2.3 Data analysis

To determine the similarity of species used between the indigenous groups, the Sørensen–Dice coefficient formula was used:

QS = *a*/[*a* + 0.5 (*b* + *c*)]

where QS is the quotient of similarity, which ranges from 0 (no similarity) to 1 (identical), *a* is the number of shared species, and *b* + *c* is the number of species in samples *b* + *c*.

## 3. Results

A total of 57 interviews were conducted with the targeted respondents from the three indigenous groups of interest. Each interview lasted approximately 30–120 minutes.

Of the 57 respondents, 20 were Kedayans, 20 were Brunei Malays, and 17 were Lun Bawangs. Table 1 shows the number of respondents interviewed per category for each indigenous group.

### 3.1 Pregnancy, childbirth, and postpartum care in Brunei

Upon confirmation of pregnancy, women in Brunei Darussalam, regardless of ethnic group, are encouraged to go for regular check-ups at the Maternal and Child Health clinics closest to their homes throughout the antenatal period. When pregnant mothers are ready to deliver, they are usually admitted to a hospital where the birth will be professionally handled by skilled healthcare personnel or midwives. Nurses or an immediate family member are often permitted to accompany the mother to comfort her and ease her tension during delivery. Subsequent postnatal check-ups are then offered to mothers at the Maternal and Child Health clinics, in addition to child healthcare, postpartum health recovery, and health education services (Ministry of Health Brunei, 2011). Nevertheless, traditional postpartum care and rituals are also commonly practised by a number of ethnic groups to enhance postnatal recovery from the potential trauma that may come with childbirth.

### 3.2 Plants used by the three indigenous groups

**Figure 2.**
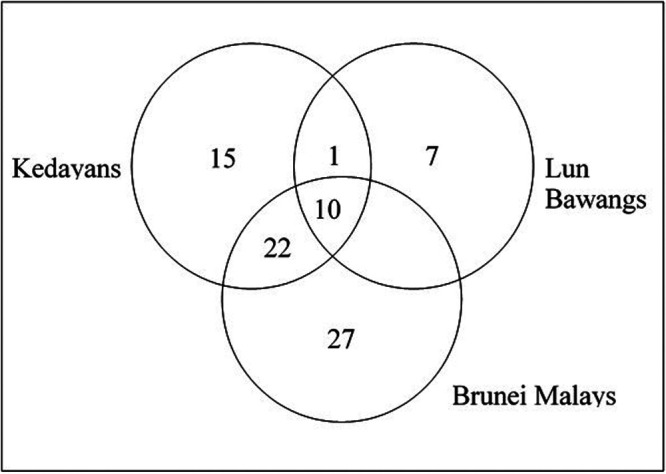
Venn diagram depicting the number of plant species commonly used by the indigenous groups for postpartum treatment.

Our study revealed a total of 82 plant species, belonging to 39 different families, used by the Bruneian Malay, Kedayan, and Lun Bawang indigenous groups for various postpartum applications. In particular, 59 medicinal plants have been identified for traditional Malay postpartum treatments, whereas 48 and 18 plants were recorded for the Kedayan and Lun Bawangs, respectively. Table 2 lists the details of the plants that were reportedly used by the three indigenous groups, their applications and the plant parts used. Among the medicinal plants recorded, 10 were commonly used by all three indigenous groups. Figure 2 summarizes the number of plants commonly incorporated into the traditional postpartum rituals of the three indigenous groups studied herein.

Table 3 shows the QS for the common plant species between the tribes. A high QS of 0.374 was recorded between the Kedayans and Brunei Malays, which indicated that the plants used by these two indigenous communities for postpartum treatment are of high similarity. The Kedayans and Lun Bawangs scored a QS of 0.250, followed by the Brunei Malay and Lun Bawangs, which showed the lowest QS of 0.206, signifying the lowest similarity among the tribes.

### 3.3 Traditional postpartum practices among the three indigenous groups

#### 3.3.1 Confinement period

According to village elders and practitioners from the Kedayan, Brunei Malay, and Lun Bawang groups, postnatal mothers are advised to carry out a period of confinement at home. The confinement period is commonly known as *berpantang* among the three indigenous groups. During this time, the mothers are prohibited from leaving their homes because they are believed to be vulnerable to ‘evil spirits’ or ‘wind’ that would infiltrate their body and cause illnesses. The heightened vulnerability of mothers during the postpartum period may be due to *nifas* (bleeding after childbirth), which can attract ‘evil spirits’ that can bring harm and cause illness to the mother if encountered.

The elderlies, practitioners, traditional healers, and postnatal caregivers from various villages claimed that the main purpose of postnatal confinement is to ensure that the mother is properly rested and her health recovered following pregnancy and childbirth. Furthermore, home confinement and traditional postpartum rituals are believed to help prevent postnatal depression in mothers.

Confinement periods practised by the Lun Bawangs range from a month to 40 days. However, some Lun Bawangs claimed that they do not abide by any specific confinement period. Both Kedayans and Malays adopted a 44-day confinement period. A Kedayan elder and traditional healer explained that the rationale behind the specific number of confinement days for the Kedayans and Malays stemmed from their belief that childbirth causes damage to 44 branches of veins that can only be repaired with proper rest for the corresponding number of days.

During the period of confinement, the new mothers are taken care of by their own mothers or an experienced elderly woman from their family or her husbands’ family. Some mothers, especially the older generation of Lun Bawangs, would carry out their own postpartum treatments with herbs and medicines they have prepared in advance prior to child delivery. The Kedayan and Malay community, on the other hand, usually employ traditional postnatal caregivers on certain days to perform special treatments such as herbal baths to boost postpartum recovery. Such traditional postnatal herbal treatments, including herbal baths and massages, are increasingly available in local modern spa facilities and are popular among young mothers residing in the urban areas.

In addition to taking herbal or steam baths, herbal decoction washes, and traditional body massages, the Bruneian postpartum tradition, typically of the Malay community, also observes confinement in a warm room as part of the postpartum treatment. It is crucial for the mother to keep her body warm and prevent the ‘invasion of wind’ during her confinement period. Thus, it is also regarded taboo or *pantang* to consume certain foods that can induce flatulence or ‘wind,’ such as cabbage, cucumber, and long beans, during this period. The mothers are required to obey strict dietary prescriptions that only permit food with ‘warming’ properties which are described as produce grown under the hot sun that are sweet, spicy, sodium-rich, and high in fat content. Herbal decoctions and preparations with this property, such as *jamu*, are largely consumed during this critical stage.

Postpartum treatments during confinement also include restoration of the uterus and the figure of the mother to her pre-pregnancy condition. Additional measures are also taken to aid the expulsion of lochia from the uterus as it may otherwise cause excruciating pain for the mother. Lochia is referred to as *sembatu* among the Malays and Kedayans, although the term *darah tajam* (sharp blood) is also commonly used among the three indigenous groups. During the confinement, it is imperative that the mother follows the taboos laid out by the elderly practitioners. All the three indigenous groups observe the *pantang* of refraining from sexual relations with the husband throughout the confinement period.

#### 3.3.2 ‘Mother roasting’

It is a common practice in Brunei to keep the body of a woman warm over the course of a few weeks immediately after giving birth. This practice is known as ‘roasting’ and the method varies between each of the three indigenous groups. It is a common belief among the Malays, Kedayans, and Lun Bawangs that the practice of roasting is to constrict the orifices of childbirth and, at the same time, to prevent the ‘cold’ and ‘wind’ from entering the vulnerable mother, restore the uterus to its pre-pregnancy condition, and alleviate postpartum abdominal pain. Additionally, it is also claimed to shrink veins that are believed to have become swollen during childbirth, speed up expulsion of lochia, and stop the bleeding. According to an elderly couple, the Malays believe that roasting can also alleviate pain and therefore any parts of the body that are sore, tender, or aching can be warmed up by the heat during the roasting process.

In Brunei, roasting is referred to as *berdiang* by all three of the indigenous groups in this study. The roasting ritual is carried out by burning charcoal or dried coconut husks in a fireplace built by the husband, the mother of the woman who has just given birth, or any family member who has had the experience of building a fireplace for the practice. The fireplace is usually of an appropriate size to warm up the mother from the waist down. It is usually situated in the woman’s bedroom or a room designated for the purpose of ‘mother roasting.’ In the olden days, Kedayans and Malays would make use of any big wooden trunk, which they called *kayu belaman*, to produce the heat. Nowadays, the younger generations prefer using electric heaters for roasting instead of old-fashioned fireplaces.

Traditionally, a wooden bed called a *kalangkapa* is also used by Kedayan, Malay, and Lun Bawang mothers. In the past, when the Lun Bawangs used to reside in stilt houses, a mirror would be suspended in the space beneath the house directly under the *kalangkapa* which is believed to deter ghosts. Nowadays, a lime (*Citrus aurantifolia* (Christm.) Swingle) is placed next to the mother’s bed instead for the same purpose.

In general, Lun Bawang, Kedayan, and Malay mothers undergo roasting for a duration between two weeks to 40 days. Roasting may be carried out twice a day—from dawn until 12 noon, and then again from 8 pm until midnight. However, the timing can be adjusted to accommodate the preference of the mother. According to the Kedayan tradition, it is advisable to allow the fire to burn continuously.

Hot massage stones are also used in combination with the practice of roasting. Smooth flat stones are first heated on a fire and then wrapped in a cloth or leaves of *Dillenia suffruticosa*, known locally as *simpor*. It is mainly applied to the back or stomach, or placed at each side of the body when the mother is laying down. This is practised by the Lun Bawangs and the Kedayans. However, the Kedayans use bricks in place of massage stones.

#### 3.3.3 Herbal baths and steam baths

Herbal baths and steam baths are two main ways of cleansing in the postpartum practices of the Kedayan, Malay, and Lun Bawang cultures. In some circumstances, steam baths are carried out inside a tent made from straw mats. As for herbal baths, hot water infused with a mixture of plants is used. Some common plants used by the three indigenous groups include *Aidia borneensis* (*sambah bagangan*), *Blumea balsamifera* (L.) DC (Ngai camphor or *sambung*), *Cerbera odollam* Gaertn. (*berbadak* or *pong-pong*), *Citrus aurantifolia* (key lime or *limau kapas*), *Cordyline fruticosa* (L.) A. Chev. (*linjuang*), *Flemingia strobilifera* (L.) W.T. Aiton (wild hops or *ringan-ringan*), *Gendarussa vulgaris* Nees (*serimbangun hitam/putih*), *Leucosyke capitellata* Wedd. (*balik sumpah*), *Lygodium microphyllum* (Cav.) R. Br. (*ribu-ribu*), *Macaranga* *beccariana* Merr. (*sedaman layang*), *Mallotus macrostachyus* (Miq.) Müll. Arg. (*balik angin*), *Melastoma malabathricum* L. (*kuduk-kuduk*), *Securidaca innappendiculata* Hassk. (*langir*) and *Vitex pinnata* L. (*kulimpapa*).

For all three indigenous groups, mothers are advised not to bathe or shower during the confinement period because it is believed that ‘wind’ might enter their bodies and cause postnatal depression. The Malays refer to the emotional upheaval caused by ‘wind’ as *duduyan*. In particular, Lun Bawangs strongly advise mothers against washing their hair during the confinement period or at least for one to three weeks because it is believed that the veins in the head are dilated after childbirth, whereby exposure of the scalp to water during the postpartum period will lead to serious headaches called *bentan*. If the mother feels the need to clean herself, she may take a hot bath or wipe her body with hot water.

Most of the Lun Bawangs stated that they do not have specific days for taking baths. However, a Lun Bawang elderly villager pointed out that they take herbal baths on the 3rd, 7th, 14th, and 40th days of confinement, which is the same as that claimed by the Malays. As for the Kedayans, herbal baths are taken on days 3, 7, 14, and 44. The respondents did not know the significance of taking herbal baths on the specific days 3, 7, 14, and 44, but it was assumed that the mother’s body would undergo major changes during those days.

On some occasions, ritual baths are also performed by postnatal caregivers. For Muslim mothers, the Islamic way of cleansing, known as the *ghusl* bath (ritual bath) or in Malay, *mandi wiladah*, will be performed on the 7th and 40th days postpartum. The ritual involves the recitation of specific prayers followed by the act of cleansing or bathing by the caregiver on the mother and ablution according to Muslim rites.

#### 3.3.4 Massage

According to tradition, Kedayan and Malay mothers undergo postpartum massages after the ritual baths on the 3rd, 7th, 14th, and 40th days post-delivery, and on the 3rd, 7th, 14th, and 44th days of confinement, respectively. In contrast, the Lun Bawangs believe that it is safer to carry out massages on mothers one or two weeks after delivery.

The massages are performed by experienced postnatal caregivers. The whole body of the mother will be massaged; however, the area surrounding the uterus is given more emphasis and will be massaged slowly and with gentle pressure. The purpose of the massage is to return the uterus to its pre-pregnancy size and position, and to ameliorate the removal of the lochia. It is believed that the womb sags as a result of pregnancy and childbirth, and may cause the woman to suffer chronic loin, pelvic, and lower abdominal pain. Thus, the massages are claimed to be beneficial as a preventive measure against these symptoms.

#### 3.3.5 Stomach binding (berbarut)

Stomach binding, or also known as *berbarut*, is a common postpartum practice in all three indigenous cultures. For this practice, a piece of cloth is wrapped around the stomach of the mother to flatten and tighten the abdomen, and prevent panniculus after pregnancy. The mothers will have their stomachs bound according to their own tradition for a period of 40 days or throughout the confinement period to achieve optimum results.

In general, a mixture of *Citrus aurantifolia* (key lime) and calcium carbonate is first applied to the stomach and then secured with a binding cloth around the abdominal region. The Lun Bawangs sometimes apply heated *Piper betle* L. (betel or *sirih*) leaves to their stomachs before wrapping with the binding cloth. For the Kedayans and Malays, a mixture of herbal ingredients called *luta* is also prepared and applied to the abdomen before binding. The composition of *luta* differs between the Kedayans and Malays, albeit for the same purpose. Specifically, the mixture prepared by the Malays consists of grounded rhizomes of *Curcuma longa* (turmeric), *Curcuma xanthorrhiza* (Javanese ginger, locally known as *temulawak*), *Alpinia galanga* (galangal or locally known as *lengkuas*), *Cinnamomum verum* (cinnamon), *Myristica fragrans* (nutmeg), *Piper retrofractum* (Javanese long pepper), dried *Aloe vera* extract, and a spice that is locally known as *ketumbong*. On the other hand, the Kedayans use the rhizome of *Zingiber officinale* (ginger), leaves of *Spondias dulcis* (June plum, locally known as *kedondong*), leaves of *Morus* sp., leaves of *Piper betle*, rind of *Garcinia parviflora* (Brunei cherry, locally known as *asam aur aur*) fruit and *Piper nigrum* L. (black pepper) to prepare the *luta*. Additionally, prior to wrapping the stomach with a binding cloth, the Kedayans fasten an additional black cotton belt (*tali gintung*) around the woman’s waist to lift up the womb and prevent sagging.

#### 3.3.6 Topical applications

Other than for massages and binding of the stomach, the Bruneian Malay community also observes the use of other topical treatments for postpartum care. One of them is referred to as *urap*, which is a kind of lotion constituted from *Curcuma longa* L. (turmeric), *Garcinia parviflora* Benth. (Brunei cherry or *asam aur aur*), *Cocus nucifera* L. (coconut) oil, and salt. This mixture is applied all over the body to restore veins that have become swollen during pregnancy. Another topical treatment is in the form of a poultice called *pilis*. Essentially, this comprises ground *Curcuma longa* L. (turmeric) and it is rubbed onto the forehead to prevent dizziness. Other than the Malays, none of the other two indigenous groups from this study incorporate such topical applications in their postpartum treatments.

#### 3.3.7 Dietary prescriptions and restrictions (pantang)

Following childbirth, women are traditionally perceived to be in a ‘cold’ state, prompting recommendations to consume foods and beverages that are considered ‘hot’ in nature, while avoiding those classified as ‘cold.’ Some of the common ‘cold’ vegetables that are regarded as taboo for all three indigenous groups to consume during the confinement period include water spinach, spinach, long beans, cucumber, cabbage, *Stenochlaena palustris* (jungle fern), and eggplants. Other ‘cold’ fruits such as watermelon are also to be avoided to prevent feverish symptoms (or *kambuh* according to the Malays and Kedayans) and to ensure the mothers stay warm. The avoidance of ‘cold’ food is also to prevent stomach discomforts or aches in breastfed babies because they would be susceptible to the ‘wind’ that may arise from the ‘cold’ food consumed by the mothers.

Cold water is also prohibited because it is believed to cause coagulation of the lochia which may retard its expulsion from the body and cause pain. Additionally, foods rich in allergens, such as yam, prawns, and marine fishes, are also to be avoided because they have been associated with malodourous lochia discharge. The Lun Bawang mothers also avoid spicy food and coffee because they believe the breastfed babies may be affected.

Specifically for the Lun Bawangs, freshwater fish, chicken, or beef will have to be first dried or barbecued before they are added into soups for postpartum mothers. They are recommended to only consume meat two or three months after delivery to prevent high blood pressure, especially for mothers over the age of 40. Lun Bawang mothers often consume Chinese wine soups with ginger to replenish the ‘warmth’ in their bodies. Furthermore, Lun Bawangs, as well as Malays, also take a kind of dried smoked fish called *tahai* cooked in soup with ginger, salt, and *Garcinia parviflora* (Brunei cherry) as a side dish with rice to promote lactation.

Chicken or cow bones are important ingredients in the preparation of postpartum recovery meals for the Kedayans. Chicken soups are exclusively prepared with free-range chicken or *ayam kampung* due to its low fat composition. Kedayan mothers also consume the shoots of the *Averrhoa bilimbi* L. (*belimbing*), *Carica papaya* L. (papaya), *Ocimum tenuiflorum* L. (holy basil or *kemangi*) and also *Centella asiatica* (L.) Urb. (Asian pennywort or *pengaga*) after giving birth for general health. The shoot of the *Vitex pinnata* L. (*kulimpapa*) and *Cosmus caudatus* Kunth (*rancah-rancah* or *ulam raja*) are also believed to be beneficial for healing internal and external wounds that have resulted from childbirth.

#### 3.3.8 Herbal drinks

Lun Bawangs, Kedayans, and Malays consume a number of herbal drinks that are claimed to restore energy, speed up lochia removal, and keep the body warm. All three indigenous groups consume a decoction called *aing kunyit*, or turmeric drink, during postpartum recovery to heal internal wounds, warm and restore the uterus condition, accelerate lochia removal, and prevent foul-smelling lochia discharge. However, the recipe varies between the different groups. For the Lun Bawangs, *aing kunyit* is usually prepared with *Curcuma longa* L. (turmeric), *Garcinia parviflora* Benth. (Brunei cherry or *asam aur aur*), *Piper betle* L. (betel or *sirih*), and honey. Nevertheless, some Lun Bawangs eat the turmeric directly, or consume a water mixture of just the crushed turmeric. The drink is taken 4–7 days after delivery for a period of 14–40 days. The same ingredients are also used by the Kedayans; however, palm sugar is used instead of honey, with the addition of *Zingiber officinale* Roscoe (ginger) and salt. As for the Malays, *Garcinia parviflora* Benth. (Brunei cherry or *asam aur aur*), *Piper betle* L. (betel or *sirih*), *Zingiber officinale* Roscoe (ginger), salt, and palm sugar are also used but they would also incorporate *Kaempferia galanga* Linn. (aromatic ginger or *cekur*), *Zingiber zerumbet* (L.) Roscoe ex Sm. (bitter ginger or *lempuyang*), and *Piper nigrum* L. (black pepper). However, some Malay traditional practitioners may prepare the turmeric drink with only *Curcuma longa* L. (turmeric), *Garcinia parviflora* Benth. (Brunei cherry or *asam aur aur*), *Piper betle* L. (betel or *sirih*), honey, and salt. In particular, the Kedayans and Malays claimed that the turmeric concoction needs to be prepared with leaves that have laterally arranged veins for optimum effect. They also insist on regular consumption of ‘*aing kunyit*’ immediately after childbirth.

In addition to *aing kunyit*, the Kedayans and Malays also consume other drinks including *irup-irupan*, *marjum*, and *pupuk*. The ingredients and recipes for each drink differ between the two groups. In particular, the Malays have three variations of *irup-irupan*, known as *irup-irupan peparam* or *irup-irupan No. 1* or *irup-irupan carah*, *irup-irupan No. 2*, and *irup-irupan banglai*. The Malays incorporate spices imported from India, whereas the Kedayans make use of more local plants collected from the forest. The Kedayans believe that *irup-irupan* and *marjum* can warm up the body, expel lochia, and also condense the uterus. For the Malays, *irup-irupan* serves as a tonic to improve the health of the mother, warm her body, improve blood circulation, expel lochia, and prevent malodourous vaginal discharge. Specifically, *irup-irupan banglai* is taken to improve the health of the mother and prevent sagging of the breasts. *Marjum* is also said to improve blood circulation, revitalize the body of the mother, and recover her health and strength. It is claimed that *marjum* prevents *carah*, which is a state of exhaustion as a result of energy-draining activities such as childbirth, characterized by sunken eyes, loss of weight, and poor response to medication. These drinks are also believed to prevent *uri* or postnatal depression. Meanwhile, *pupuk* is taken by both the Kedayans and Malays to warm up the body, expel lochia, and also to contract the uterus and vagina. Furthermore, the Kedayans also consume a decoction of the stems and leaves of *Vitex pinnata* L. (*kulimpapa*) which are boiled in water for the purpose of expelling lochia and to prevent foul discharges. These drinks are recommended to be taken regularly throughout the day.

In particular for the Lun Bawangs, most mothers also consume *aing kandis*, which is a type of Chinese herb-infused wine. This is not particularly practised by the Kedayans and Malays due to their religious belief. The Lun Bawangs believe that the medicinal wine can keep the new mothers warm and improve the discharge of lochia.

According to Malay culture, mothers are recommended to drink a concoction of raw eggs from free-range chicken, honey, and *Piper nigrum* L. (white pepper) immediately after birth. The concoction is claimed to boost energy and complete lochia excretion within a week. Although not compulsory, the Malays would also consume *aing masak 40 hari* (40th day herbal decoction) daily for about a week. This drink is made up of 23 different Indian spices (including four unidentified species—*sipetir*, *ketumbong*, *cangkok*, and *patah tulang*), *Usnea barbata* f. *elegans* (Stirt.) anon. (synonym), *Alpinia galanga* L. (galangal or *lengukuas*), and *Curcuma xanthorhizza* Roxb. The spices and herb mixture are cooked in 5 litres of water until the decoction is reduced to 4 litres. The amount of water that is consumed from the decoction is replaced and the decoction is re-boiled daily until the decoction goes bland. These dietary consumptions of raw egg mixtures and ‘*aing masak 40 hari*’ are unique to the Malays as they are not observed to be consumed by the Kedayans and Lun Bawangs.

#### 3.3.9 Breast care

After giving birth, some women experience breast engorgement due to clogged milk ducts. The engorgement is often painful and can cause fevers. To relieve the tension, the Lun Bawangs place a piece of *Brassica oleracea* (cabbage) or warm towel on the engorged breasts. This is believed to reduce the pain, unclog the milk ducts, and promote milk flow. The hot towel practice is also similarly carried out by the Kedayans and Malays. In addition, Malay mothers also use the bristle of a comb that has been immersed in hot water to brush or massage the breast in an outward motion to stimulate milk flow.

To increase milk production, Kedayans and Lun Bawangs tend to avoid solid food and only consume liquid foods such as soups and porridge. As mentioned before, Lun Bawangs and Malays consume tahai soup cooked with ginger, salt, and *Garcinia parviflora* (Brunei cherry) with rice to promote lactation. The Kedayans and Malays are also encouraged to take the vegetable, *Portulaca oleracea* (*langiruh*), to increase milk production. Particular to the Kedayan beliefs, excess milk should be discarded into the fireplace where roasting is done to avoid evil spirits from licking the milk as it is believed to cause a condition called *palau susu* in which the woman develops pus on her breasts.

#### 3.3.10 Healing of the delivery wounds and removal of stitches

Delivery of babies often involves perineal laceration and stitches. Therefore, rapid recovery is required to avoid prolonged discomfort in the mother. The Lun Bawangs, Kedayans, and Malays encourage mothers to sit in warm saltwater baths for about 10 minutes daily for the first week after delivery to accelerate the healing process. Additionally, the Lun Bawangs would add *Blumea balsamifera* (Ngai camphor) to the warm saline bath for enhanced effects. This practice may have been adopted from the Chinese because it is also a common practice among Chinese to use this plant for healing perineal tears and stitches.

For the Malays, a decoction called *pembasuhan* is also prepared to expedite wound healing and dissolve/remove the stitches faster. The decoction is prepared with a mixture of *Zingiber purpureum* Roscoe (Cassumunar ginger or *banglai*) rhizomes, *Terminalia chebula* (*majalawi*/*kadakai*/*manjaputeri*) fruits, *Curcuma longa* (turmeric) rhizomes, *Areca catechu* L. (betel nut or *pinang*) fruits, *Croton caudatum* (*manjakani*), aluminium potassium sulfate salt (alum, or *tawas* in Malay), and *Acacia catechu* (L.f.) Willd., Oliv (*kachu*) bark. This preparation can be used by the mother every time she cleans her wounds.

### 3.4 Behavioural taboos during the postnatal period

There are several taboos observed by the three indigenous groups throughout the postpartum period to avoid slow recovery, complications, and undesirable ailments during old age. The Lun Bawangs have an especially strong belief in such postnatal taboos.

Lun Bawang mothers are prohibited from going outdoors at night for the first two weeks following childbirth because they are believed to be highly susceptible to colds during this period. Sleeping under a mosquito net is also taboo because it is traditionally believed to cause blindness. Furthermore, mothers are also advised not to fall asleep too soundly because it is feared that deep sleep would cause the mothers to suffer partial or complete blindness before the age of 40.

On the other hand, similar taboos are observed by the Kedayans and Malays. While carrying out home confinement after childbirth, hard labour and exposure to rain is highly discouraged for the mothers. The mothers must sit with proper posture with their calves closely placed against each other. The mothers are also not allowed to walk hastily or recklessly because it is believed to cause displacement of the uterus and impede recovery of the veins that may have ruptured during childbirth. It is also common to have a few black peppercorns bound to their big toes by a piece of black cloth to keep the body warm and prevent fever. Additionally, bounded toes are said to prevent mothers from accidentally kicking their big toes on walls or doors as bleeding may be delayed especially during the postpartum period. The black cloth must be worn throughout the confinement period and retied if loose.

### 3.5 Trends among mothers of younger generation

A number of young mothers from each of the three indigenous groups expressed their preference for over-the-counter herbal drinks that were imported from neighbouring countries, such as Malaysia and Indonesia, over local herbal tonics that have to be prepared fresh by themselves. Other than convenience, the imported herbal tonics were also claimed to be more palatable compared to the local preparations which can be quite spicy. The traditional local tonics were also claimed to cause constipation due to the ‘heaty’ or ‘warm ingredients that they contain. The young mothers also believed that the modern variations are more efficacious at restoring their body and health to pre-pregnancy conditions.

## 4. Discussion

Postpartum care is a quintessential biocultural arena in which physiological vulnerability intersects with culturally codified notions of womanhood, kinship, and cosmology (Wiley and Allen, 2020). Building on Ellen’s (2006) ethnobiological insights, scholars now routinely regard local knowledge systems—especially plant‑based pharmacopoeias and ‘hot–cold’ taxonomies—as legitimate, dynamic forms of science rather than residual folklore. This biocultural‑ethnobiological perspective therefore provides a valuable framework for interpreting Bruneian practices and for situating them within wider anthropological debates on adaptation, embodiment, and medical pluralism.

Our study revealed that traditional rituals and practices for postpartum care persist among the three indigenous groups in Brunei despite modern allopathic healthcare being widely available throughout the country. This shows that traditional practices are still valued by the members of the community. These knowledge and practices have been passed down for generations and are believed to be effective therapeutic treatments for mothers and newborns. However, the knowledge of the traditional practices is mostly confined to older people above the age of 50. Most of the younger generations lack the interest to learn about traditional practices and are mostly pursuing these practices out of respect or at the insistence of the elders from their families. This is a common situation, which is also observed in the comprehensive study on the Chinese *zuòyuèzi* (‘doing-the-month’) practice which revealed how women tactically navigate psychological stressors, generational expectations, and biomedical advice—often hybridizing ritual with personal comfort needs. Findings from a 13-study meta-synthesis show that women either tolerate, negotiate, or actively reshape confinement rules, echoing the approach taken by young Bruneian mothers who outsource traditional massages to urban spas or substitute local tonics with branded imports (Xin et al., 2024). Urban, highly educated Chinese mothers likewise report cognitive dissonance between ‘modern science’ and inherited prescriptions, yet still uphold certain practices for fear of long-term debility—a motivation likewise articulated by Bruneian informants regarding ‘*duduyan*’ and ‘*sembatu*.’

Our study showed that all three indigenous groups generally regard the postpartum period as a period of utmost significance, as do many from other Southeast Asian countries including Indonesia, the Philippines (Siregar et al., 2021), Laos (Lamxay et al., 2011), and Thailand (Kamdee and Nuntaboot, 2021). The mothers from all three indigenous groups undergo home confinement for a maximum of 44 days and abide by a set of cultural rituals and customs to regain their energy and strength as well as to restore their pre-pregnancy health and figure. The confinement period offers a chance for new mothers to acclimate to their new role while they recover physically, mentally, and emotionally from childbirth. It also provides an opportunity to further enrich the bond between the mother and child. Similar to the beliefs of the indigenous mothers interviewed in this study, the traditional practice of postpartum confinement or home rest have been reported to deter mothers from developing postnatal depression in the Chinese culture (Guo et al., 2021). Interestingly, the spiritual reasoning behind practice of seclusion as a precautionary measure to protect mothers and newborns from malevolent entities was also a common belief in rural Bangladesh (Jahan and Islam, 2024).

During the postpartum recovery period, the indigenous people emphasized the importance for mothers of staying warm such as by ‘roasting’ (see section 3.3.2) and taking baths/washing with warm herbal solutions only on specific days (see section 3.3.3) to regulate ‘heat’ and ‘wind.’ On the contrary, mothers from some Western countries were encouraged to take baths as soon and often as possible to maintain personal hygiene (Vo and Desai, 2021). To our knowledge, there have not been any studies conducted to evaluate the significance of the traditional practices of staying warm and reducing exposure to water in postpartum care and treatment. The rationale behind the practice of restricting water contact during the postpartum period may have arisen from past experience of their ancestors who had faced sanitary issues with water as a source of potential infections. Nevertheless, recent ethnographies continue to reveal the perceived benefits of warming or introducing heat to the postpartum body which reputedly prevented or treated various postpartum issues including body aches, weakness, lactation issues, lochia, uterine contractions, loss of appetites, and disrupted sleep (Bazzano et al., 2020; Fu et al., 2023; Lubis et al., 2024). These accounts mirror the belief of the Bruneian Kedayan ‘roasting’ rituals, suggesting the practice as a form of biocultural adaptation as it underscored the shared logic of restoring vascular integrity and overall wellbeing of the mother across different cultures.

In addition to staying warm via roasting and herbal baths, the three indigenous groups also emphasized the avoidance of ‘cold’ foods, including certain vegetables and fruits, during postpartum recovery because these foods are believed to affect the mothers’ health, as well as the baby’s through breastfeeding. Additionally, the mothers were also advised to take herbal tonics laden with spices. The consumption of postpartum herbal tonics is a ubiquitous practice in many Southeast Asia countries (Silalahi et al., 2020). Spices are generally considered ‘hot’ and would thus benefit the mother’s postpartum recovery as most spices possess body-warming properties (Yahya et al., 2023). Physiologically, the warming effect of some ‘hot’ spices or food can be felt as the skin would perspire and a warm sensation can be felt in the mouth or throughout the body after consuming them. The customary practice of avoiding ‘cold’ foods during the postpartum period is an integral part of the Malay cultural system (Yahya et al., 2023). Traditionally, mothers are strongly recommended to consume ‘warm’ foods to restore the balance and energy in their bodies, which have become extremely ‘cold’ following excessive energy drainage and blood loss during childbirth. This was also a common notion for other Asian cultures such as in Cambodia, China, and Indonesia, where warming the postpartum body via dietary or other physical means was integral especially in their early stages of postpartum recovery (Bazzano et al., 2020; Fu et al., 2023; Lubis et al., 2024). Nevertheless, contemporary healthcare practices have advised mothers against restricting their intake of ‘cold’ foods which includes fruits and vegetables as they are important nutrient sources for postpartum women (Yeh et al., 2014). Instead, it is recommended that mothers consume a balanced diet comprising a healthy portion of a variety of food.

According to our study, there were several similarities especially between the traditional postpartum practices and herbal plants used by the Kedayan and Brunei Malay indigenous groups. This is possibly a result of frequent interaction between the indigenous groups in the past, which led to the exchange of knowledge between them. The traditional postpartum treatments of the indigenous groups seem to have some Indian and Chinese influences because Ayurvedic and traditional Chinese medicine elements can be recognized in indigenous practices such as extensive use of natural products such as spices in the preparation of herbal or medicinal tonics for postpartum recovery (Vaamonde et al., 2022). The use of spices in the preparation of herbal drinks was also observed in other Southeast Asian countries such as Malaysia (Appalasamy et al., 2022), Indonesia (Muslichah et al., 2021), and Thailand (Krungkraipetch and Kwanchainon, 2023).

In comparison, the use of spices is less common among the Kedayan community as compared to the Brunei Malays. Instead, the Kedayans tend to incorporate more plants sourced from the forest in their postpartum treatment and diets. The Kedayans used to reside in the peripheries of the water village and are well known for their wisdom about the jungle and forest products. Traditionally, they hunt animals in the forests and were the main suppliers of forest and agricultural products to the Brunei Malays due to their familiarity with the forest. Therefore they are more accustomed than the Malays to consuming forest plants, either raw or cooked, as vegetables.

As for the Lun Bawang, who mostly reside in the Temburong district, they had frequent interactions with the Chinese and also Malays who lived there. This explains the Chinese and Malay influences and the extensive use of Chinese medicine in their traditional postpartum treatment.

Nevertheless, the common postpartum practices of confinement, including ‘mother roasting,’ baths, massages, stomach binding (*berbarut*), dietary prescriptions and restrictions (*pantang*), herbal drinks, breast care, care of delivery wounds, and removal of stitches, were commonly observed among the three local indigenous groups. This shared tradition may be due to the opening up of previously inaccessible rural areas as this facilitates mobility and interdistrict migration. Intercultural marriages between the indigenous groups may have also led to the exchange of knowledge between communities. Additionally, government resettlement schemes and employment opportunities in urban areas may have further contributed to the phenomenon due to increased interaction between the once-isolated groups.

With globalization, the dissemination of modern knowledge and ideas has become rapid, and has had direct consequences for traditional beliefs and practices of many cultures, including those of the Brunei Malays, Kedayans and Lun Bawangs. For instance, in Brunei, many young mothers opt for imported postpartum care products from neighbouring countries, such as Malaysia and Indonesia, due to the convenience and effectiveness that the products have been marketed to have. Because the Malays from Brunei, Malaysia, and Indonesia share similar cultures, it is easy for Bruneians to adopt the practices from the bordering nation. Unfortunately, this has led to a decline in local indigenous practices.

To combat the issue of diminishing local indigenous traditions, extensive research and proper documentation are crucial to preserve indigenous knowledge and know-how. Social media platforms may also provide a means to popularize the benefits of the traditional treatments and practices. The commercialization of imported postpartum care products has shown success in garnering the attention and interest of younger generations, and thus is also likely to be a viable method to preserve and promote indigenous Bruneian tonics and decoctions. Furthermore, local indigenous treatment practices may also be modernized to appeal more to young mothers such as by offering traditional herbal baths and massages in modern spa facilities.

Ethnographic critiques collected in *Anthropologies of Global Maternal and Reproductive Health* highlight how global policy scripts on ‘skilled birth attendance’ frequently collide with local birthing ontologies and midwifery authority (Wallace et al., 2022). Parallel tensions are now visible in Brunei: while universal health coverage ensures hospital deliveries, postpartum recovery remains a pluralistic domain navigated between state clinics, spa entrepreneurs, and village elders. Calls for a ‘global culture of respectful maternity care’ urge policymakers to recognize such pluralism and embed cultural competence in postpartum programming as squarely aligned with the preservation and innovation approach suggested herein (Puthussery et al., 2023).

## 5. Conclusion

Despite widespread access to allopathic medicine and healthcare in the country, traditional postpartum treatment practices remain an important part of the Malay, Kedayan, and Lun Bawang indigenous communities of Brunei Darussalam. From our study, it is evident that most of the knowledge of traditional postpartum rituals is confined within the group of medical practitioners or caregivers who are above 50 years of age because the younger generation have generally shown less interest in learning about traditional ways of postpartum care or therapy.

Our study revealed common practices between the three indigenous groups in terms of postpartum care, especially between the Kedayans and Malays due to religious and cultural similarities. The mutual knowledge and beliefs between the groups can be attributed to the migration from the once-isolated traditional villages to resettlement areas that comprise neighbourhoods with diverse ethnicities and cultures. Intercultural marriages between the indigenous groups may also contribute to the common postpartum practices noted in this study. The practices of the Lun Bawangs have relatively fewer similarities with the other two indigenous groups, probably due to differences in religion and cultural influences.

The traditional postpartum practices of the Brunei indigenous communities are unique and of great heritage importance and value. However, this knowledge is constantly challenged by rapid modernization. To preserve the authenticity of the customs and beliefs, it is imperative that extensive research and proper documentation is conducted. Other means to popularize these indigenous postpartum practices, such as offering herbal tonics and treatment services in the form of commercialized products, are also vital to ensure that the traditional knowledge remains relevant and applicable in modern times.

## Acknowledgments

This study was supported by Universiti Brunei Darussalam.

We wish to thank Dr Saiful Islam from the Anthropology, Faculty of Arts and Social Sciences, Universiti Brunei Darussalam for his advice and guidance on the sociological aspect of the study.

## Funding Statement

This research did not receive any specific grant from funding agencies in the public, commercial, or not-for-profit sectors.

## Ethics Approval Statement

Ethical clearance was obtained prior to conducting the study (REF: IBER/RA/30).

## Conflicting Interests

The authors declare that there are no conflicts of interest.

## Author Contributions

M.A. contributed to the conceptualization, data curation, result analysis and interpretation, manuscript preparation, and review of the final manuscript. G.M.P.Y. contributed to the analysis and interpretation of results, manuscript preparation, visualization, manuscript editing, and review of the final manuscript. S.R.S., M.M.A. and A.N. contributed to the conceptualization, supervision, analysis and interpretation of results, and review of the final manuscript.

## Figures and Tables

**Table 1.** Number of respondents per category for each of the indigenous groups interviewed

**Respondents**	**Parents**	**Traditional healer**	**Postnatal caregiver**	**Elderly**
**Kedayans**	9	2	3	6
**Brunei-Malays**	8	0	1	11
**Lun Bawangs**	9	1	0	7

**Table 2.** Plants used by the indigenous tribes in their traditional postpartum treatments

**Scientific name**	**Family**	**Vernacular name**	**Uses in postpartum treatment**	**Part used**	**Indigenous users**
*Acacia catechu* (L.f.) Willd.	Leguminosae	*Kachu*	Herbal drinks	Heartwood/bark	Malays
*Acorus calamus* L.	Araceae	*Jerangau*	Herbal drink	Rhizomes	Malays
*Aidia racemosa* (Cav.) Tirveng.	Rubiaceae	*Sambar bagangan*	Herbal bath (leaves), herbal drink (roots boiled with the root of *Mallotus macrostachyus* to shrink the veins)	Leaves, roots	Malays and Kedayans
*Allium cepa* L.	Liliaceae	*Bawang merah*	Herbal drink	Rhizomes	Malays
*Anethum graveolens* L.	Umbelliferae	*Das manis*	Herbal drink (to shrink uterus, revive energy, and warm up body)	Seeds	Malays
*Aloe vera* Mill.	Xanthorrhoeaceae	*Jadam*	Herbal drink	Leaves	Malays
*Alpinia galanga* Willd.	Zingiberaceae	*Langkuas*	Herbal drink (to shrink uterus, revive energy, and warm up body)	Rhizomes	Malays and Kedayans
*Areca catechu* L.	Arecaceae	*Pinang*	Topical application (to remove stitches)	Fruits	Malays
*Averrhoa bilimbi* L.	Oxalidaceae	*Belimbing*	Eaten raw (for general health after birth)	Leaves	Kedayans
*Blumea balsamifera* (L.) DC	Asteraceae	*Sambung*	Herbal bath (to reconnect veins)	Leaves	Malays, Kedayans, and Lun Bawangs
*Boesenbergia rotunda* (L.) Mansf.	Zingiberaceae	*Tamu kunci*	Herbal drink (to shrink uterus, revive energy, and warm up body)	Rhizomes	Malays and Kedayans
*Brassica oleracea* L.	Brassicaceae	*Kubis*	Topical application (to reduce breast pain)	Leaves	Lun Bawangs
*Caesalpinia sappan* L.	Leguminosae	*Kayu Sappang*	Herbal drink	Wood	Malays
*Carica papaya* L.	Caricaceae	*Kepayas*	Eaten raw (for general health after birth)	Leaves	Kedayans
*Centella asiatica* (L.) Urb.	Umbelliferae	*Penggaga*	Eaten raw (for general health after birth)	Leaves	Kedayans
*Cerbera odollam* Gaertn.	Apocynaceae	*Berbadak*	Herbal bath	Leaves	Malays and Kedayans
*Cinnamomum verum* J. Presl.	Lauraceae	*Kayu manis*	Herbal drink (to shrink uterus, revive energy, and warm up body)	Tree bark	Malays
*Citrofortunella microcarpa* (Bunge) Wijnands	Rutaceae	*Limau kasturi*	Herbal bath	Leaves	Kedayans
*Citrus aurantifolia* (Christm.) Swingle	Rutaceae	*Limau kapas*	Herbal bath/topical application (mixture for slimming)	Leaves/fruits	Malays, Kedayans, and Lun Bawangs
*Citrus hystrix* DC	Rutaceae	*Limau purut*	Herbal bath	Leaves	Lun Bawangs
*Citrus maxima* (Burm.) Osbeck	Rutaceae	*Limau besar*	Herbal bath	Leaves	Lun Bawangs
*Cordyline fruticosa* (L.) A. Chev.	Liliaceae	*Linjuang*	Herbal bath	Leaves	Malays, Kedayans, and Lun Bawangs
*Coriandrum sativum* L.	Umbelliferae	*Ketumbar*	Herbal drink (to shrink uterus, revive energy, and warm up body)	Fruits	Malays and Kedayans
*Cocos nucifera* L.	Arecaceae	*Kelapa*	Herbal drink	Seed kernels	Malays
*Cosmos caudatus* Kunth	Compositae	*Rancah-rancah cina*	Eaten raw (to heal wound)	Leaves	Kedayans
*Croton caudatum*	Euphorbiaceae	*Manjakani*	Herbal drink (to shrink uterus)	Fruits	Malays
*Cryptocarya massoia* (Oken) Kosterm.	Lauraceae	*Masoori*	Herbal drink	Tree bark	Malays
*Cuminum cyminum* L.	Umbelliferae	*Jintan putih*	Herbal drink (to shrink uterus, revive energy, and warm up body)	Fruits	Malays and Kedayans
*Curcuma caesia* Roxb.	Zingiberaceae	*Kunyit hitam*	Herbal drink (to shrink uterus, revive energy, and warm up body)	Rhizomes	Kedayans
*Curcuma heyneana* Valeton & Zijp	Zingiberaceae	*Tamu giring*	Herbal drink (to shrink uterus, revive energy, and warm up body)	Rhizomes	Kedayans
*Curcuma longa* L.	Zingiberaceae	Kunyit	Herbal drink (to cleanse blood, remove bad odours of the blood, and heal internal wounds)Topical application (mixture for slimming/lotions applied to minimize swollen veins)	Rhizomes	Malays, Kedayans, and Lun Bawangs
*Curcuma* sp.1	Zingiberaceae	*Tamu kuning*	Herbal drink (to shrink uterus, revive energy, and warm up body)	Rhizomes	Malays and Kedayans
*Curcuma* sp.2	Zingiberaceae	*Tamu hijau*	Herbal drink (to shrink uterus, revive energy, and warm up body)	Rhizomes	Malays and Kedayans
*Curcuma xanthorrhiza* Roxb.	Zingiberaceae	*Tamu lawak*	Herbal drink (to shrink uterus, revive energy, and warm up body)	Rhizomes	Malays and Kedayans
*Curcuma zedoaria* (Christm.) Roscoe	Zingiberaceae	*Tamu putih*	Herbal drink (to shrink uterus, revive energy, and warm up body)	Rhizomes	Malays and Kedayans
*Cymbopogon citratus* (DC) Stapf	Gramineae	*Serai*	Herbal bath	Leaves	Lun Bawangs
*Cymbopogon nardus* (L.) Rendle	Gramineae	*Serai wangi*	Herbal bath	Leaves	Kedayans and Lun Bawangs
*Dillenia suffruticosa* (Griff.) Martelli	Dilleniaceae	*Simpur*	Topical application (to wrap hot stones on the stomach)	Leaves	Lun Bawangs
*Elettaria cardamomum* (L.) Maton	Zingiberaceae	*Pelaga*	Herbal drink	Fruits	Malays
*Euodia latifolia* DC	Rutaceae	*Badak*	Herbal bath	Leaves	Malays
*Flemingia strobilifera* (L.) W. T. Aiton	Leguminosae	*Ringan-ringan*	Herbal bath	Leaves	Malays, Kedayas, and Lun Bawangs
*Foeniculum vulgare* Mill.	Umbelliferae	*Das pedas*	Herbal drink (to shrink uterus, revive energy, and warm up body)	Seeds	Malays
*Garcinia parviflora* Benth.	Clusiaceae	*Asam aur-aur*	Herbal drink (to cleanse blood, remove bad odours of the blood, and heal internal wounds) Topical application (mixture for slimming/lotions applied to minimize swollen veins)	Rind of the fruit	Malays, Kedayans, and Lun Bawangs
*Gendarussa vulgaris* Nees	Acanthaceae	*Serimbangun hitam*/*serimbangun putih*	Herbal bath	Leaves	Malays, Kedayans, and Lun Bawangs
*Hibiscus rosa-sinensis* L.	Malvaceae	*Bunga raya*	Herbal bath	Leaves	Lun Bawangs
*Kaempferia galanga* L.	Zingiberaceae	*Cakur*	Herbal drink (to shrink uterus, revive energy, and warm up body)	Rhizomes	Malays and Kedayans
*Kaempferiae rotunda* L.	Zingiberaceae	*Kunyit putih*	Herbal drink (to shrink uterus, revive energy, and warm up body)	Rhizomes	Kedayans
*Leucosyke capitellata* Wedd.	Urticaceae	*Balik sumpah*	Herbal bath	Leaves	Malays and Kedayans
*Lygodium microphyllum* (Cav.) R. Br.	Schizaeaceae	*Ribu-ribu*	Herbal bath	Leaves	Malays, Kedayans, and Lun Bawangs
*Macaranga beccariana* Merr.	Euphorbiaceae	*Sedaman layang*	Herbal bath	Leaves	Malays and Kedayans
*Mallotus macrostachyus* (Miq.) Müll. Arg.	Euphorbiaceae	*Balik angin*	Herbal bath (leaves) Herbal drink (roots boiled with the root of *Aidia racemosa* (Cav.) Tirveng. to shrink the veins)	Leaves, roots	Malays and Kedayans
*Melastoma malabathricum* L.	Melastomaceae	*Kuduk-kuduk*	Herbal bath	Leaves	Malays and Kedayans
*Mimosa pudica* L.	Leguminosae	*Daun semalu*	Herbal bath/topical application (mixture for slimming)	Leaves	Kedayans
*Morinda citrifolia* L.	Rubiaceae	*Mengkudu*	Herbal bath	Leaves	Kedayans
*Morus alba*	Moraceae	*Karakatau*	Topical application (mixture for slimming)	Leaves	Kedayans
*Myristica fragrans* Houtt.	Myristicaceae	*Buah pala*	Herbal drink (to shrink uterus, revive energy, and warm up body)	Fruits	Malays
*Nigella sativa* L.	Ranunculaceae	*Jintan hitam*	Herbal drink (to shrink uterus, revive energy, and warm up body)	Seeds	Malays
*Ocimum tenuiflorum* L.	Lamiaceae	*Kemangi*	Eaten raw (for general health after birth)	Leaves	Kedayans
*Pandanus amaryllifolius* Roxb.	Pandanaceae	*Pandan*	Herbal bath	Leaves	Lun Bawangs
*Phaleria macrocarpa* (Scheff.) Boerl.	Thymelaeaceae	*Mahkota dewa*	Topical application (mixture for slimming)	Leaves	Kedayans
*Peucedanum japonicum* Thunb.	Umbelliferae	*Ghanti*	Herbal drink	Root	Malays
*Pimpinella anisum* L.	Umbelliferae	*Jintan manis*	Herbal drink (to shrink uterus, revive energy, and warm up body)	Seeds	Malays and Kedayans
*Piper betle* L.	Piperaceae	*Sirih*	Herbal drink/topical application (for slimming)	Leaves	Malays, Kedayans, and Lun Bawangs
*Piper cubeba* L.f.	Piperaceae	*Kumukus*	Herbal drink	Fruits	Malays
*Piper nigrum* L.	Piperaceae	*Lada sulah hitam/putih*	Herbal drink/topical application (*lada sulah hitam*: tied to big toe to prevent excessive bleeding and fever; *lada sulah putih*: mixture for slimming)	Seeds	Malays and Kedayans
*Piper retrofractum* Vahl	Piperaceae	*Cabi dagang*	Herbal drink	Fruits	Malays
*Portulaca oleracea* L.	Portulacaceae	*Langiruh*	Eaten as vegetables (to promote lactation)	Leaves	Malays and Kedayans
*Santiria apiculata A.W. Benn.*	Burseraceae	*Sambar burung*	Herbal bath	Leaves	Kedayans
*Saussurea lappa* Clark.	Asteraceae	*Puchok*	Herbal drink	Root	Malays
*Securidaca innappendiculata* Hassk. Ssp. Innappendiculata (*S. tavoyana* A.W. Benn.)	Polygalaceae	*Langir*	Herbal bath	Root	Malays and Kedayans
*Spondias dulcis Parkinson*	Anacardiaceae	*Kedundung*	Topical application (mixture for slimming)	Leaves	Kedayans
*Sindora wallichii* Benth.	Leguminoceae	*Sipetir*	Herbal drink	Seeds	Malays
*Syzgium aromaticum* (L.) Merr. & Perr.	Myrtaceae	*Cengkih*	Herbal drink	Seeds	Malays
*Terminalia chebula* Retz.	Combretaceae	*Kadakai/majalawi/manjaputeri*	Herbal drink	Fruits	Malays
*Trachyspermum copticum* (L.) Link	Umbelliferae	*Musi*	Herbal drink	Fruits	Malays
*Trigonella foenum-graecum* L.	Leguminosae	*Halba*	Herbal drink	Seeds	Malays
*Vitex pinnata* L.	Lamiaceae	*Kulimpapa*	Herbal bath	Leaves	Malays and Kedayans
*Woodfordia fruticosa* Kurz.	Lythraceae	*Sudu wayah*	Herbal drink	Flower	Malays
*Zingiber officinale* Roscoe	Zingiberaceae	*Halia*	Herbal drink/topical application (mixture for slimming)	Rhizomes	Malays, Kedayans, and Lun Bawangs
*Zingiber officinale* Var. *rubrum Theilade*	Zingiberaceae	*Halia merah/Halia bara*	Herbal drink (to shrink uterus, revive energy, and warm up body)	Rhizomes	Malays and Kedayans
*Zingiber purpureum* Roscoe	Zingiberaceae	*Banglai*	Herbal drink/topical application (mixture for slimming)	Rhizomes	Malays and Kedayans
*Zingiber zerumbet* (L.) Roscoe ex Sm.	Zingiberaceae	*Lempuyang*	Herbal drink	Rhizomes	Malays

**Table 3.** Quotient of similarity (QS) for plant species between tribes

**Compared groups**	**QS**
**Kedayans and Brunei Malay**	0.374
**Brunei Malay and Lun Bawangs**	0.206
**Kedayans and Lun Bawangs**	0.250
